# Mechanisms and Genetic Drivers of Resistance of Insect Pests to Insecticides and Approaches to Its Control

**DOI:** 10.3390/toxics13080681

**Published:** 2025-08-16

**Authors:** Yahya Al Naggar, Nedal M. Fahmy, Abeer M. Alkhaibari, Rasha K. Al-Akeel, Hend M. Alharbi, Amr Mohamed, Ioannis Eleftherianos, Hesham R. El-Seedi, John P. Giesy, Hattan A. Alharbi

**Affiliations:** 1 Applied College, Center for Bee Research and Its Products, King Khalid University, Abha 61413, Saudi Arabia; 2 Department of Biology, Faculty of Science, University of Tabuk, Tabuk 71491, Saudi Arabia; nmohammad@ut.edu.sa (N.M.F.); aalkhaibari@ut.edu.sa (A.M.A.); 3 Department of Zoology, Faculty of Science, King Saud University, Riyadh 11451, Saudi Arabia; ralogial@ksu.edu.sa; 4 Department of Biology, College of Science, Princess Nourah bint Abdulrahman University, Riyadh 11671, Saudi Arabia; hemalharbi@pnu.edu.sa; 5 Department of Entomology, Faculty of Science, Cairo University, Giza 12613, Egypt; 6 Department of Biological Sciences, The George Washington University, Washington, DC 20052, USA; ioannise@gwu.edu; 7 Department of Chemistry, Faculty of Science, Islamic University of Madinah, Madinah 42351, Saudi Arabia; halsaeedi@iu.edu.sa; 8 Department of Environmental Science, Baylor University, Waco, TX 76798-7266, USA; jgiesy@aol.com; 9 Department of Veterinary Biomedical Sciences and Toxicology Centre, Western College of Veterinary Medicine, University of Saskatchewan, Saskatoon, SK S7N 5B3, Canada; 10 Department of Integrative Biology and Center for Integrative Toxicology, Michigan State University, East Lansing, MI 48824, USA; 11 Department of Plant Protection, College of Food and Agriculture Sciences, King Saud University, Riyadh 11451, Saudi Arabia; halharbii@ksu.edu.sa; 12 Chair of Date Palm Research, College of Food and Agriculture Sciences, King Saud University, Riyadh 11451, Saudi Arabia

**Keywords:** insecticide resistance, molecular mechanisms, metabolic detoxification, target-site resistance, emerging mechanisms, cross-resistance, insecticide resistance management (IRM)

## Abstract

The escalating challenge of resistance to insecticides among agricultural and public health pests poses a significant threat to global food security and vector-borne disease control. This review synthesizes current understanding of the molecular mechanisms underpinning resistance, including well-characterized pathways such as target-site mutations affecting nicotinic acetylcholine receptors (nAChRs), acetylcholinesterase (AChE), voltage-gated sodium channels (VGSCs), and γ-aminobutyric acid (GABA) receptors, and metabolic detoxification mediated by cytochrome P450 monooxygenases (CYPs), esterases, and glutathione S-transferases (GSTs). Emerging resistance mechanisms are also explored, including protein sequestration by odorant-binding proteins and post-transcriptional regulation via non-coding RNAs, such as microRNAs (miRNAs) and long non-coding RNAs (lncRNAs). Focused case studies on *Aedes aegypti* and *Spodoptera frugiperda* illustrate the complex interplay of genetic and biochemical adaptations driving resistance. In *Ae. aegypti*, voltage-gated sodium channel (VGSCs) mutations (V410L, V1016I, F1534C) combined with metabolic enzyme amplification confer resistance to pyrethroids, accompanied by notable fitness costs and ecological impacts on vector populations. In *S. frugiperda*, multiple resistance mechanisms, including overexpression of cytochrome P450 genes (e.g., *CYP6AE43*, *CYP321A8*), target-site mutations in ryanodine receptors (e.g., I4790K), and behavioral avoidance, have rapidly evolved across global populations, undermining the efficacy of diamide, organophosphate, and pyrethroid insecticides. The review further evaluates integrated pest management (IPM) strategies, emphasizing the role of biopesticides, biological control agents, including entomopathogenic fungi and parasitoids, and molecular diagnostics for resistance management. Taken together, this analysis underscores the urgent need for continuous molecular surveillance, the development of resistance-breaking technologies, and the implementation of sustainable, multifaceted interventions to safeguard the long-term efficacy of insecticides in both agricultural and public health contexts.

## 1. Introduction

The ongoing arms race between humans and insect pests remains heavily dependent on synthetic insecticides, despite mounting resistance and growing concerns about their impacts on human health and biodiversity [1,2]. The widespread and often indiscriminate use of these chemicals has accelerated resistance, posing serious threats to ecosystems, public health, and agriculture. Insect pests not only inflict extensive damage on crops, forests, stored products, and animal feed, but also serve as vectors for diseases caused by viruses, bacteria, and parasites [1,2,3,4,5]. Their impact extends beyond direct yield loss, posing serious threats to food security, disrupting ecological balance, and imposing substantial economic burdens—especially in low- and middle-income countries. Global assessments estimate yield losses of approximately 21.5% for wheat, 30% for rice, 22.5% for maize, 17% for potatoes, and 21% for soybeans, with the highest losses concentrated in food-insecure regions [3]. Moreover, the economic cost of invasive insect pests alone exceeds US$70 billion annually worldwide, with associated health impacts surpassing US$6.9 billion [6]. Since its invasion of Africa in 2016, the fall armyworm (*Spodoptera frugiperda*) has spread to over 70 countries, causing annual maize yield losses of 8.3–20.6 million tons and economic damages of US$2.5–6.2 billion [7,8].

Globally, about 3.7 million tons of pesticides were applied in agriculture in 2022, with insecticides accounting for roughly 29.5% [9]. China alone used an estimated 1.4 million tons of insecticides, according to FAOSTAT data [9,10]. These pesticides primarily target insect physiological systems, including the nervous system and energy metabolism. While chemical control has driven significant productivity gains since the Green Revolution, it has also accelerated the evolution of resistance in many pest species.

**Figure 1:**
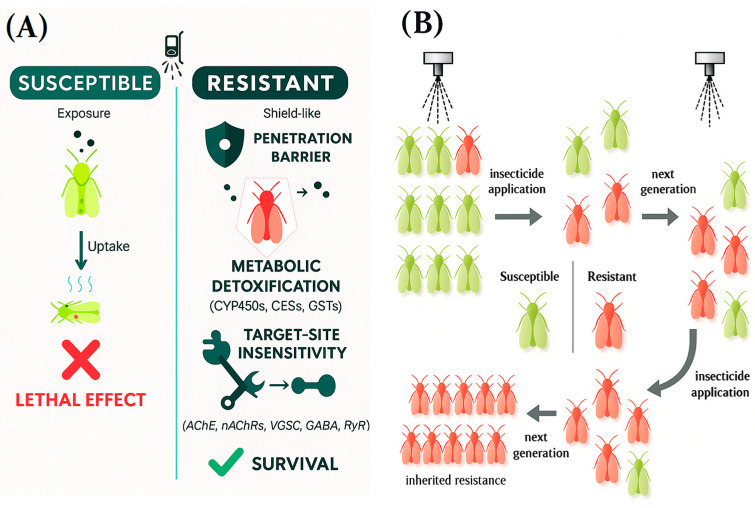
Conceptual illustration of insecticide resistance in insects. (**A**) The diagram contrasts insecticide-susceptible insects (**left**) with insecticide-resistant insects (**right**). Susceptible individuals are killed upon exposure to insecticides, whereas resistant individuals possess one or more adaptive mechanisms that enable survival. These mechanisms include reduced cuticular penetration (limiting insecticide entry), enhanced metabolic detoxification, and target-site insensitivity (e.g., mutations at the insecticide’s site of action). (**B**) Insecticide resistance emerges when these adaptive traits allow certain individuals to survive exposure to otherwise lethal doses, resulting in the selection, persistence, and eventual spread of resistant populations.

While organophosphates, pyrethroids, and neonicotinoids have historically exhibited strong insecticidal efficacy, over 600 arthropod species have developed resistance to these compounds, with thousands of rigorously documented cases worldwide. Resistance to insecticides occurs when pests inherit reduced sensitivity, causing pesticides to fail repeatedly even when applied as directed [5]. The Insecticide Resistance Action Committee (IRAC) describes resistance as “a heritable change in the sensitivity of a pest population that is reflected in the repeated failure of a product to achieve the expected level of control when used according to the label recommendation for that pest species” [11]. Resistance not only leads to failure in controlling insect pests but also necessitates higher dosages and the use of newer—often more toxic—chemical agents, thereby compounding environmental and health concerns [12]. Figure 1 illustrates the process of resistance development.

Resistance is driven by various factors, including genetic variability, high reproductive rates, and operational practices such as repeated use of similar chemical classes [13]. These factors facilitate rapid selection of resistant phenotypes and increase the likelihood of resistance allele fixation within populations. Furthermore, globalization and trade have enhanced the spread of resistant pests across borders, turning localized problems into global challenges. This relentless selection pressure underscores resistance as an “*evolutionary arms race*” demanding disruptive solutions. Traditional chemical-centric approaches are outpaced by pest adaptation, necessitating paradigm-shifting strategies grounded in evolutionary theory—such as evolutionary rescue interventions or gene drives targeting resistance alleles—to break the cycle of resistance escalation [14].

These evolutionary pressures give rise to two major categories of resistance mechanisms: (1) target-site resistance, involving structural mutations at insecticide binding sites (e.g., [15]); and (2) metabolic resistance, characterized by enzymatic degradation of insecticides before they reach their site [5]. Other less common but increasingly recognized mechanisms include behavioral resistance, where pests modify their actions to avoid exposure, and cuticular resistance, involving changes in the insect cuticle that reduce insecticide penetration. Understanding these diverse mechanisms is critical for designing countermeasures and delaying the onset of resistance.

Considering the growing challenge of resistance to insecticides, there is an urgent need to review and evaluate existing pest management paradigms. Reliance on chemical controls alone is no longer tenable. A shift toward integrated pest management (IPM), which combines biological control agents, cultural practices, resistant crop varieties, and judicious use of chemicals, is imperative. Such an approach not only ensures long-term sustainability but also mitigates the adverse effects of chemical overuse on ecosystems and human health [16].

This review provides an examination of the molecular and biochemical foundations of resistance to insecticides, drawing attention to key genes and pathways involved. It also evaluates the limitations of conventional chemical control and explores integrated alternatives aimed at sustainable pest suppression. By consolidating current knowledge and identifying emerging trends, this review aims to support the development of informed, adaptable, and ecologically sound pest management strategies.

## 2. Key Mechanisms Underlying Insecticide Resistance

Resistance to insecticides arises through four main mechanisms. Behavioral resistance involves altered behaviors like outdoor resting or oviposition avoidance, offering low to moderate resistance but delaying physiological resistance. Penetration resistance results from thickened or modified cuticles, reducing insecticide uptake and providing moderate, cross-class protection. Target-site insensitivity includes mutations at binding sites (e.g., *kdr*, *Ace-1*, *Rdl*) and leads to high, class-specific resistance and rapid control failure. Metabolic resistance, the most potent, is driven by overexpression or amplification of detoxifying enzymes (P450s, esterases, GSTs), resulting in high, broad-spectrum resistance and cross-resistance to multiple insecticide classes [17,18] (Table 1). These mechanisms often act in combination.

### 2.1. Molecular Mechanisms of Target-Site Resistance

Target-site resistance arises when specific mutations alter the molecular conformation of an insecticide’s binding site, diminishing or abolishing its affinity. This prevents the compound from exerting its intended neurotoxic or biochemical effect. Resistance mechanisms can be classified into four broad categories based on the nature of the target site: (1) neurological targets (e.g., AChE, nAChR, sodium channel, chloride channel, etc.); (2) physiological and metabolic targets (e.g., chitin synthase, ryanodine receptor, mitochondrial respiratory chain, etc.); (3) growth and developmental targets (e.g., juvenile hormone receptors, ecdysteroid receptors, etc.); and (4) other novel or rare targets (e.g., octopamine receptor, midgut cell membranes, oxidative phosphorylation and decoupling agents, etc.).

#### 2.1.1. Neurological Targets

##### Nicotinic Acetylcholine Receptor (nAChR) Mutations

Neonicotinoids, acting as high-affinity agonists of insect nAChRs, have become one of the most widely used classes of insecticides globally due to their systemic properties, potency, and relatively favorable mammalian toxicity profile [31]. In insects, nAChRs are pentameric ligand-gated ion channels composed of various combinations of α- and β-subunits. The neonicotinoid-binding site is formed at the subunit interface, involving loops A–C of the α-subunit and loops D–E of the β-subunit. Among these, residues in loop D of the β1 subunit play a critical role in neonicotinoid recognition and sensitivity [32].

Target-site resistance has emerged in several pest species through point mutations that impair neonicotinoid binding without disrupting acetylcholine-mediated neurotransmission. In *Myzus persicae*, two such substitutions in the β1 subunit—R81T and V101I—markedly reduce neonicotinoid binding affinity while maintaining receptor function [15,33]. These mutations confer high-level resistance to multiple compounds, including imidacloprid, thiamethoxam, and dinotefuran. All tested homozygous R81T clones (81TT) exhibited markedly higher resistance to imidacloprid and thiacloprid compared to other genotypes, while heterozygous clones (81RT) displayed slightly elevated but overlapping resistance levels with wild-type clones. This pattern is consistent with semi-recessive inheritance, with mean dominance coefficients of 0.316 for imidacloprid and 0.351 for thiacloprid [34]. While the R81T mutation alone confers substantial resistance, bioassays demonstrate that co-occurrence with elevated *CYP6CY3* expression further amplifies resistance via synergistic interaction between target-site and metabolic mechanisms [35].

Radioligand binding assays revealed a complete loss of the high-affinity [^3^H]-imidacloprid binding site and altered low-affinity interactions in the R81T variant, leading to a marked reduction in overall binding affinity. This effect is likely attributable to the disruption of key electrostatic interactions at the R81 residue [15]. Computational modelling supports this mechanism, demonstrating a substantial decrease in binding affinity associated with the R81T substitution [36]. In thrips, mutations V65I and V104I in the β1 subunit of nAChRs contribute to neonicotinoid resistance by reducing receptor binding to neonicotinoids and sulfoxaflor (V65I) and by increasing neonicotinoid efficacy while decreasing agonist affinity (V104I) [37]. Similarly, in *Bemisia tabaci*, dual mutations (A58T&R79E) in the BTβ1 nAChR subunit confer target-site resistance to neonicotinoids, with molecular modeling suggesting a mechanism driven by electrostatic repulsion at the orthosteric site [38]. In *Musca domestica*, reduced expression of the *nAChR α*2 subunit has been implicated in neonicotinoid resistance, particularly when coupled with enhanced detoxification via CYPs [39]. In *Thrips palmi*, the nAChR α1 subunit serves as the primary target of neonicotinoids, exhibiting higher agonist affinity and efficacy compared to the α2 subunit [40]. Collectively, these studies underscore the importance of nAChR subunit variation in neonicotinoid resistance, albeit through distinct molecular mechanisms.

Spinosad, a macrocyclic lactone insecticide, targets a distinct nAChR subunit—α6 (Dα6)—and exhibits a different mode of action from neonicotinoids. In *Drosophila melanogaster*, a CRISPR/Cas9-engineered G275E substitution in Dα6 produces a ~66-fold decrease in spinosad sensitivity, confirming the causality of this mutation even in the absence of changes in other subunits [41]. Resistance to spinosad tends to be recessively inherited and polygenic, contrasting with the more dominant neonicotinoid resistance conferred by β1 mutations.

Sulfoxaflor, a sulfoximine insecticide, acts as an nAChR agonist but targets a different receptor subtype from neonicotinoids. Its mode of action involves sustained activation of nAChRs, leading to hyperexcitation, paralysis, and eventual death. Despite its chemical distinctiveness, sulfoxaflor remains toxic to non-target pollinators [42]. Resistance has been documented in multiple pest species—including *Aphis gossypii*, *Nilaparvata lugens*, and *B. tabaci*—and is primarily associated with overexpression of CYPs, although target-site mutations have also been implicated [43,44,45]. Partial cross-resistance with neonicotinoids has been observed, emphasizing the need for integrated resistance management.

Field studies of 35 *M. persicae* colonies across Europe revealed a strong correlation between resistance ratios to sulfoxaflor and imidacloprid (Pearson’s *r* = 0.939, *p* < 0.0001). However, this correlation was absent in Spanish colonies carrying the R81T mutation (*r* = 0.2901, *p* = 0.3604), indicating that R81T alone does not confer altered sulfoxaflor susceptibility [46]. Notably, synergist bioassays using PBO significantly reduced—but did not eliminate—resistance in a single field-collected R81T-harboring clone (FRC), with residual resistance factors of 234-fold for imidacloprid and 26-fold for thiamethoxam following PBO treatment. These results demonstrate that the R81T mutation alone confers substantial resistance, independent of metabolic detoxification pathways [15].

The FRC clone, collected from southern France in 2009, was homozygous for R81T and exhibited concurrent *CYP6CY3* gene amplification [15]. Experimental studies showed that sustained absence of neonicotinoid selection did not alter nAChR protein levels in the R81T-harboring *M*. *persicae* FRC strain [47]. Inheritance assays confirm the semi-recessive nature of R81T, with dominance coefficients of 0.316 (imidacloprid) and 0.351 (thiacloprid). This implies slower allele fixation rates in the field, even under strong selection pressure [34].

Collectively, these findings demonstrate that specific mutations in the β1 and Dα6 subunits of nAChRs act as dominant or semi-dominant resistance mechanisms by structurally disrupting the insecticide binding interface. Table 2 provides an overview of recent nAChR mutations linked to resistance or susceptibility in major insect pests. Although such mutations often co-occur with metabolic resistance (e.g., *CYP6CY3* overexpression), other factors—such as α2 subunit downregulation in *M. domestica* and the recessive inheritance of spinosad resistance—introduce substantial heterogeneity in resistance dynamics and fitness trade-offs across species.

Future research should prioritize resolving the precise pentameric stoichiometry of α-β subunit assemblies that determine high- versus low-affinity neonicotinoid binding, as subunit composition may significantly influence receptor sensitivity. The development of high-resolution homology models or cryo-electron microscopy (cryo-EM) structures of mutated nAChRs is essential for quantifying conformational changes in ligand binding and channel gating. Additionally, ecological studies are needed to evaluate the fitness costs associated with R81T and Dα6 mutations under realistic conditions, including interspecific competition, environmental stress, and cyclic insecticide exposure. Finally, elucidating the epistatic interactions and genetic background effects—such as those involving cytochrome P450 haplotypes or nAChR gene duplications—will be critical for predicting the spread and stability of resistance in field populations.

##### Voltage-Gated Sodium Channel Alterations (Knockdown Resistance, *kdr*)

Pyrethroids and DDT target VGSCs, essential for action potential propagation in insect neurons. Resistance is conferred through mutations in the VGSC gene, commonly known as vssc1, particularly at position 1014 in domain II, where leucine is substituted with phenylalanine (L1014F) [62]. This mutation disrupts insecticide binding and prolongs channel opening, reducing neuronal excitability. *Kdr*-type mutations have been reported across multiple species, including *M. domestica*, *Cx. quinquefasciatus*, and *L. decemlineata* [63,64]. Variants *kdr* and super-*kdr* are differentiated, the latter involving additional amino acid substitutions and higher resistance levels [65]. Additionally, oxadiazine insecticides, such as indoxacarb, also target sodium channels. Resistance to indoxacarb has been reported in several insect species, with mutations in sodium channel genes identified as responsible for reduced binding affinity [66]. For instance, the presence of the F1845Y and V1848I mutations in the VGSCs of *T. absoluta* was strongly associated with indoxacarb resistance, closely correlating with the resistant phenotype [67].

#### 2.1.2. Physiological and Metabolic Targets

##### Aminobenzoic Acid Amide Insecticides (Ryanodine Receptor)

Aminobenzoic acid amide insecticides, commonly known as diamides—such as flubendiamide, chlorantraniliprole, tetrachlorantraniliprole, and cyantraniliprole—act by targeting the ryanodine receptor (RyR) in insects, disrupting calcium ion release in muscle cells and leading to paralysis and death. These compounds, including broflanilide and tetraniliprole, have shown consistently high field efficacy against *S. frugiperda* larvae and effectively suppressed *S. exigua* populations shortly after application [68,69]. Nonetheless, the development of resistance is a growing challenge. Alterations in the *RyR* gene have been associated with decreased binding affinity of these insecticides, resulting in reduced effectiveness, as reported in *P. xylostella* [70]. Moreover, research indicates that various chemical categories—such as ryanodine, phthalamides, and anthranilamides—interact with different sites on the insect RyR [71]. Computational docking studies have also identified species-specific differences in the chlorantraniliprole binding site across pests, including *Sogatella furcifera*, *Leptinotarsa decemlineata*, and *Helicoverpa assulta* [72]. These insights emphasize the importance of thoroughly understanding RyR binding mechanisms, particularly in pests like *S. frugiperda*, to elucidate resistance pathways and support the development of sustainable insecticide strategies [72].

##### Benzoylurea Insecticides (*Chitin Synthase*)

Benzoylurea insecticides (BUs), which target chitin synthase—a key enzyme in insect exoskeleton formation—remain widely used in IPM. Resistance to BUs in arthropods is primarily driven by nonsynonymous mutations in the *chitin synthase 1* gene (*CHS1*; referred to as *CHSA* in Lepidoptera), which encodes the catalytic subunit essential for chitin biosynthesis during molting. These mutations localize to transmembrane helices (TMH) 5–7 of *CHS1*, where conserved isoleucine residues are substituted to induce steric hindrance, blocking insecticide binding while preserving enzymatic function. Examples include I1040M in *S. frugiperda CHSA*, which confers resistance to lufenuron [73]; I1042M in *P. xylostella CHS1*, which causes cross-resistance to diflubenzuron and flufenoxuron [74]; homologous mutations such as I1017F in *Tetranychus urticae* and *F. occidentalis* [74,75]; and I1043F/L/M in *Culex pipiens*, which reduces diflubenzuron efficacy [76]. Emerging mutations, such as I1040T/V in *S. frugiperda*, further reduce BU binding affinity [56], demonstrating that convergent substitutions (I → M/F/L/T/V) represent a dominant evolutionary mechanism. Secondary metabolic resistance occurs via glutathione S-transferase (GST) overexpression, such as *PxGSTs1* in *P. xylostella*, which enhances BU detoxification by glutathione conjugation mediated by catalytic residues (Ser65, Tyr97), enabling π-π stacking interactions [77].

#### 2.1.3. Growth and Developmental Targets

##### Juvenile Hormone Receptors and Ecdysteroid Receptors

Insects rely on hormonal signals like juvenile hormone (JH) and ecdysteroids for normal growth and development [78]. Juvenile hormone analogs (e.g., methoprene) and ecdysteroid inhibitors (e.g., tebufenozide) are commonly used insecticides. Resistance to insecticides by juvenile hormone receptors (JHRs) and ecdysteroid receptors (EcRs) involves complex genetic adaptations, including mutations, gene overexpression, and epigenetic modifications. These adaptations enable insects to evade the effects of insect growth regulators (IGRs) and other endocrine-disrupting insecticides. For JHRs, such as Methoprene-tolerant (Met), resistance mechanisms include target-site mutations like Y129F, which impair juvenile hormone binding [79,80], gene overexpression [81], and functional redundancy between Met and its paralog germ cell-expressed (Gce) [82,83]. Additionally, post-transcriptional regulation by non-coding RNAs and RNA methylation further modulates resistance [84,85,86]. In EcRs, mutations in the ligand-binding domain [87,88,89] and alterations in receptor complex interactions, such as JH-mediated competition for Ultraspiracle (USP), disrupt 20-hydroxyecdysone (20E) signaling. This disruption affects molting and detoxification pathways [90,91,92]. Additionally, cross-talk between JH and EcR pathways modulates resistance, with JH-Met complexes inhibiting EcR dimerization and recruiting co-repressors to suppress metamorphosis [93,94,95].

#### 2.1.4. Other Novel or Rare Targets

##### Octopamine Receptors

In insects, octopamine exerts pleiotropic effects as a neurotransmitter, neuromodulator, and hormone, regulating behavior, physiology, and immunity [96,97]. Its receptors have become key targets for insecticides due to their central physiological role [98]. Resistance to octopamine receptor antagonists can arise through mutations in the receptor gene, presenting challenges for control strategies. Octopaminergic signaling is disrupted not only by synthetic antagonists but also by certain essential oils. These oils—such as eugenol, γ-terpineol, and cinnamic alcohol—block octopamine receptors, causing acute and sublethal behavioral effects in insects. Normally, octopamine binding elevates cyclic AMP (cAMP) levels, but this signaling is inhibited by these essential oil compounds. Notably, eugenol alone significantly reduces receptor binding even at low doses [99,100]. Conversely, octopamine receptor (OR) agonists—including formamidine pesticides like amitraz and chlordimeform—exert their effects by inhibiting monoamine oxidase and disrupting cAMP production, which leads to behavioral changes. These pesticides also directly bind to octopamine receptors as OR agonists, underscoring their complex biochemical and molecular mechanisms. Furthermore, OR agonists show synergistic effects when combined with other insecticide classes such as organophosphates, pyrethroids, and neonicotinoids, enhancing pest control efficacy. Consequently, they are important components of IPM programs [101,102,103,104].

##### Midgut Cell Membranes and Oxidative Phosphorylation/Decoupling Agents

Insecticides targeting midgut cell membranes or oxidative phosphorylation pathways are still being explored, with resistance often linked to mutations in ATP synthase subunits or increased expression of decoupling proteins. For example, periplocoside P (PSP) from *Periploca sepium* selectively depolarizes the apical membrane potential (V_a_) in *Mythimna separata* larvae by inhibiting V-type H^+^-ATPase, disrupting midgut ion transport similarly to Cry1Ab toxin but with a faster onset [105]. Likewise, the acetogenin squamocin from *Annona mucosa* uncouples oxidative phosphorylation in *Ae. aegypti* by inhibiting mitochondrial complex I (NADH-ubiquinone oxidoreductase) while also suppressing V-ATPase and autophagy-related genes (Atg1, Atg8), demonstrating multi-modal toxicity [106]. Resistance to these compounds may involve multiple mechanisms, including potential mutations in ATP synthase subunits (e.g., V-ATPase) or the upregulation of mitochondrial uncoupling proteins that reduce the proton gradient. While direct evidence in insects—particularly in midgut tissues—is still limited, similar uncoupling effects have been reported for certain insecticides and other bioactive agents, suggesting that mitochondrial dysfunction could contribute to resistance development [107,108,109,110,111].

#### 2.1.5. GABA Receptor Mutations and Resistance to Dieldrin (*Rdl*)

GABA-gated chloride channels are essential for inhibitory neurotransmission in insects, and mutations affecting their subunits represent critical mechanisms of resistance to insecticides. The *Rdl* gene encodes one such subunit, and substitutions at position A302—most notably A302S and A302G—have been strongly linked to resistance against cyclodienes and phenylpyrazoles [112,113]. Ala301 mutations in Rdl confer moderate resistance to phenylpyrazoles in *D*. *melanogaster*. Gly301 provides better survival than Ser301. Gene duplication and mutations in *D*. *simulans* also contribute to resistance [112]. These mutations are believed to disrupt GABA binding or reduce chloride ion conductance, thereby impairing the action of GABA-targeting insecticides [114].

In *D. melanogaster*, the A2′N mutation in the RDL GABA receptor confers substantial in vivo resistance to fipronil, likely through steric hindrance from bulkier side chains at the mutation site, which interfere with insecticide binding—even in heterozygous individuals [115]. Additionally, the A301S variant not only mediates resistance to cyclodienes and phenylpyrazoles but also induces neurophysiological compensation through the cholinergic system. This includes upregulation of AChE expression and activity, leading to cross-resistance against carbamates (e.g., propoxur) and organophosphates (e.g., dichlorvos), as well as altered expression of muscarinic acetylcholine receptors that confer resistance to non-selective muscarinic compounds such as pilocarpine and atropine [116]. Homology modeling and ligand-docking study predict that the A301S substitution narrows the RDL channel pore and significantly reduces the binding free energy of dieldrin and fipronil, providing atomistic evidence for a steric exclusion mechanism [116]. In *An. gambiae*, a T345S/M substitution co-occurs with the codon 296 allele and partially restores normal channel gating kinetics—suggesting that secondary mutations in transmembrane regions may compensate for *Rdl*-mediated dysfunction, a possibility yet to be examined in *D. melanogaster* [117].

In A301S-mutant *Drosophila*, the GABAergic toxicity of fipronil and dieldrin is less effectively synergized by pilocarpine, unlike in susceptible strains, suggesting altered muscarinic modulation of GABAergic pathways [118]. Furthermore, analogies to *ace-1* gene duplications in *Anopheles*, which buffer the fitness costs of resistance alleles, raise the possibility that structural variants—such as tandem duplications or heterogeneous *Rdl* alleles—may similarly stabilize the A301S genotype in *Drosophila* [119].

Despite extensive phenotypic evidence of persistence, comprehensive life-history studies (e.g., on larval development, adult longevity, fecundity, and competitive fitness) remain unpublished for the MD-RR strain (homozygous for the *Rdl* A301S resistance mutation). Such studies are crucial for understanding why the A301S mutation persists without reversion, contrasting with rapid genotype reversion observed in other species following relaxation of insecticide pressure [120].

Critically, resistance mechanisms rarely act in isolation. Epistatic interactions and compensatory adaptations—such as *CYPs* upregulation, mitigating the fitness costs of *kdr* alleles—contribute to polygenic resistance and adaptive resilience [121,122,123]. Future research should prioritize disentangling mechanistic hierarchies, distinguishing primary resistance drivers from secondary modifiers, to effectively disrupt resistance networks and delay further evolution of multi-class resistance.

### 2.2. Mechanisms of Metabolic Resistance: Enzymatic Detoxification Pathways

Insects frequently overcome insecticidal exposure not only through structural mutations at target sites but also via enhanced detoxification systems. Metabolic resistance is primarily conferred through the upregulation, gene amplification, or structural modification of key enzymes that neutralize or degrade insecticides before they reach their intended molecular targets. The detoxification process is now widely understood to involve three integrated phases: Phase I (oxidation, hydrolysis, or reduction), Phase II (conjugation of metabolites for excretion), and Phase III (efflux transport and/or sequestration). The major enzyme families implicated in metabolic resistance include Phase I enzymes such as esterases and CYPs, Phase II enzymes including glutathione-S-transferases (GSTs) and UDP-glucosyltransferases (UGTs), and Phase III transporters such as ATP-binding cassette (ABC) proteins [124,125,126].

#### 2.2.1. Esterases: Gene Amplification and Detoxification Efficiency

Esterases (particularly CESs) are integral to Phase I detoxification. They cleave ester bonds in insecticides such as pyrethroids, carbamates, organophosphates, and neonicotinoids, diminishing their toxicity before neuronal interference [124]. Esterase-based resistance in *M*. *persicae* is linked to two amplified carboxylesterase genes, *E4* and *FE4*. The *E4* form, associated with an A1–3 chromosomal translocation, is globally distributed, while *FE4*, typically found in the Mediterranean, lacks such rearrangements [127]. Resistant aphid clones can exhibit 5 to 11 times higher esterase gene copy numbers compared to susceptible populations, leading to significant degradation of insecticides before they reach their target. However, increased gene copy number or transcript abundance alone does not necessarily confirm a detoxification role.

Current approaches to verify enzymatic activity include in vitro metabolism assays using recombinant esterases, susceptibility shifts following RNA interference (RNAi) knockdown, and identification of metabolic products. For example, recombinant carboxylesterase E4 from *M. persicae* expressed in *Escherichia coli* hydrolyzed the carbamate insecticide carbaryl by 64% within 2.5 h and the organophosphate malathion by 80% within 1.25 h, directly confirming its catalytic detoxification capability [128]. Similarly, RNAi silencing of *LbEST1*, *LbEST2*, and *LbEST3* in the psocid *Liposcelis bostrychophila* increased mortality by 1.83–2.69-fold following malathion exposure, demonstrating in vivo detoxification roles through phenotypic reversion assays [129]. Furthermore, RNAi-mediated silencing of the carboxylesterase gene *Deg-CarE* in *Dermanyssus gallinae* significantly reduced esterase activity and restored susceptibility to β-cypermethrin in resistant mites, confirming *Deg-CarE*’s critical role in detoxification and resistance [130].

#### 2.2.2. Cytochrome P450 Monooxygenases: The Versatile Detoxifiers

Cytochrome P450 enzymes (CYPs) constitute a large, evolutionarily conserved family capable of metabolizing endogenous hormones, xenobiotics, and plant secondary compounds [131,132]. These monooxygenases catalyze oxidation reactions, including *N*-dealkylation, hydroxylation, desulfuration, and epoxidation, forming the biochemical cornerstone of insect metabolic resistance. In *M*. *persicae*, P450 enzymes (CYP6CY3 and CYP6CY4) play a key role in metabolic resistance. Elevated P450 expression correlates with resistance up to a threshold. The homozygous R81T mutation, combined with P450 overexpression, confers high neonicotinoid resistance. A synergistic interaction between P450s and R81T enhances resistance, though resistance variation across different R81T genotypes is only partially explained by P450 levels, suggesting other factors are involved [35].

In *M. domestica*, cytochrome P450 subunits *CYP6A1*, *CYP6D1*, and *CYP6D3* exhibit sex-specific overexpression, conferring resistance to multiple insecticide classes [133]. Additionally, *D. melanogaster* P450 variants like *CYP6w1* and *CG10737* mediate DDT resistance via similar overexpression patterns [134]. Synergists such as piperonyl butoxide (PBO) inhibit CYP enzymes, restoring insecticide sensitivity and emphasizing their crucial role in the evolution of resistance [135]. Beyond transcriptomic associations, the metabolism of insecticides by specific P450s has been confirmed using in vitro expression systems. For example, recombinant expression of CYP enzymes has demonstrated direct metabolism of lambda-cyhalothrin into less toxic hydroxylated metabolites [136], further affirming their functional role in resistance.

#### 2.2.3. Glutathione-S-Transferases (GSTs): Conjugation- and Sequestration-Based Resistance

GSTs are multifunctional enzymes catalyzing the conjugation of reduced glutathione (GSH) to electrophilic xenobiotics, facilitating their solubilization and excretion. These enzymes also contribute to oxidative stress mitigation, intracellular transport, and hormone biosynthesis [137]. In insects, GSTs are classified into three major families: Cytosolic GSTs (e.g., Delta, Epsilon, Omega, Sigma), Microsomal GSTs (also called MAPEGs), and Mitochondrial GSTs (Kappa, typically absent in insects) [138]. In *S*. *litura*, GSTe16 is crucial for pyrethroid detoxification and antioxidant defense. Knockdown and CRISPR/Cas9 validation confirm its role in resistance. Molecular analysis identifies key residues (Arg111, Asn122) for substrate specificity, indicating an evolutionary link between xenobiotic metabolism and oxidative stress response [139]. Allelic variation in epsilon GST genes contributes to DDT and pyrethroid resistance in *An. funestus*. Transcriptomic and genomic analyses reveal region-specific variants—such as *A17D26T158-GSTe3*, *L135H191A189-GSTe4*, and *T169S201E210-GSTe6*—that exhibit increased binding affinity for DDT and permethrin. Functional assays showed recombinant GSTs metabolize DDT (41–63%) and permethrin (13–25%), with the BN-*GSTe4* variant demonstrating significantly enhanced deltamethrin metabolism [140]. Transgenic expression in *Drosophila* confirms reduced DDT mortality in flies with selected alleles, indicating a key role in resistance [140]. These enzymes operate primarily in Phase II detoxification, neutralizing oxidized metabolites produced by CYPs and esterases. Their upregulation is often driven by gene duplication, promoter mutations, or stress-induced expression networks [137,138]. Recent findings indicate that certain GST isoforms possess high-affinity hydrophobic binding pockets that enable sequestration of insecticides such as lambda-cyhalothrin, thereby reducing their bioavailability and interaction with neural targets. This detoxification-by-sequestration mechanism—distinct from enzymatic conjugation—has been experimentally confirmed in *Cydia pomonella* as a key factor contributing to resistance, where exposure to sublethal doses of lambda-cyhalothrin upregulated 17 GST genes, and recombinant CpGSTd3 exhibited the highest binding activity despite no detectable metabolites [141].

#### 2.2.4. UDP-Glucosyltransferases (UGTs): An Overlooked Phase II Component

UDP-glucosyltransferases (UGTs) are increasingly recognized as critical Phase II detoxification enzymes in insects. They conjugate UDP-sugars to hydrophobic xenobiotics, enhancing their water solubility for excretion. Recent studies have identified overexpressed UGTs in resistant populations of *B. tabaci*, *H. armigera*, *S. litura*, *Anopheles funestus*, and parasitoid wasps, with direct involvement in pyrethroid and neonicotinoid resistance [142,143,144,145,146]. Functional assays using recombinant UGTs and RNAi-based knockdown approaches further confirm their metabolic roles.

#### 2.2.5. ABC Transporters: Phase III Toxin Efflux Systems

Phase III detoxification involves the active export of both conjugated and unmodified toxins from insect cells, primarily mediated by ATP-binding cassette (ABC) transporters [147]. These transporters play a critical role in regulating toxin bioavailability by facilitating efflux from midgut epithelial cells, Malpighian tubules, and other tissues. Members of the ABC transporter family, such as ABCB1, ABCG4, and ABCC2, have been implicated in resistance to various insecticides, including spinosad, pyrethroids, and *Bacillus thuringiensis* (*Bt*) toxins [126]. Their overexpression is often induced by sublethal insecticide exposure and is associated with enhanced excretory capacity, forming an important defense mechanism against toxin accumulation [147]. Experimental evidence supports the functional role of these transporters in resistance to insecticides. In *A. gossypii*, RNAi targeting ABCC2 or ABCG15 significantly reduced thiamethoxam efflux and increased insecticide susceptibility by 2.3–3.5-fold [73]. Similarly, in *T. urticae*, abamectin resistance was reversed using beauvericin (BEA), a known ABC transporter inhibitor. Co-application of BEA with abamectin led to a 4.4–7.7-fold reduction in LC_50_ values compared to abamectin alone. Molecular docking studies further confirmed BEA’s high affinity for ABCC and ABCG substrate-binding sites, indicating that it competitively inhibits xenobiotic transport [148].

## 3. Emerging and Underexplored Mechanisms of Resistance of Insects to Insecticides

Recent advances have revealed novel classes of resistance to insecticides that extend beyond conventional metabolic and target-site models. Two such mechanisms, protein-based sequestration and RNA-mediated posttranscriptional regulation, are reshaping understanding of insect adaptation under chemical pressure.

### 3.1. Sequestration Resistance: Repurposing Olfactory Proteins as Insecticide Buffers

The concept of sequestration resistance is that insects co-opt chemosensory proteins to sequester insecticides, reducing their bioavailability and toxicity. Traditionally recognized for their role in the detection of odors, odorant-binding proteins (OBPs) and chemosensory proteins (CSPs) have been co-opted to function as molecular “sponges” for xenobiotics. Overexpression of these proteins in cuticular tissues, particularly the legs, creates localized sequestration zones that limit insecticide penetration and interaction with target sites [149,150]. For example, *An. gambiae* expresses SAP2, a sensory appendage protein that can bind with deltamethrin. Resistance collapses upon SAP2 silencing via RNAi [151]. In the brown planthopper, NIOBP3 binds the neonicotinoid nitenpyram, with RNAi-mediated silencing restoring susceptibility [152]. Results of studies with transgenic *Drosophila* further confirm functional sequestration activity across multiple species, including aphids, moths, and honeybees [153,154]. These findings support the hypothesis that olfactory proteins have evolved multifunctional roles, acting as frontline defenders against insecticide exposure [155].

### 3.2. RNA-Mediated Resistance: Noncoding RNAs as Posttranscriptional Regulators

The concept of RNA-mediated resistance is based on long noncoding RNAs (lncRNAs) and microRNAs (miRNAs) that orchestrate changes in the expression of genes that enhance resistance phenotypes. Noncoding RNAs modulate the expression of genes through various mechanisms, including mRNA stabilization, miRNA sponging, and epi-transcriptomic modification. In *P. xylostella*, the lncRNA *lnc-GSTu1-AS* protects *GSTu1* transcripts from miRNA (miR-8525-5p)-mediated degradation, sustaining GST-based detoxification and chlorantraniliprole resistance [156]. In *Drosophila*, *lnc19419* acts as a competing endogenous RNA, binding miR-944 and derepressing CPCFC—a cuticular protein gene implicated in malathion resistance [157]. Epi-transcriptomic modifications such as m6A methylation in the 5’UTR of *CYP4C64* enhance gene expression and enzymatic detoxification efficiency in whiteflies [84]. These findings underscore the sophistication of RNA-based regulatory networks in modulating resistance beyond static genetic changes.

### 3.3. Role of the Microbiome in Detoxification: A Dynamic Evolutionary Defense

Insect gut microbiomes play a vital role beyond nutrition by acting as adaptive biochemical filters that metabolize insecticides into less toxic compounds, enhancing host survival and contributing to chemical resilience across diverse taxa [158,159,160]. These microbial communities dynamically respond to pesticide exposure through upregulation of degradative enzymes and shifts in community composition, often working synergistically with host detoxification systems [161,162]. Notably, symbionts play a key role in shaping the functional diversity and ecological dynamics of these interactions. This symbiotic detoxification represents an evolving defense strategy with implications for sustainable pest and pollinator management [163].

#### Role of the Microbiome in Insect Detoxification and Resistance: Mechanistic Insights

Members of the insect microbiome, including both gut-associated bacteria and intracellular endosymbionts, can influence resistance to insecticides through multiple mechanisms, not only by directly metabolizing xenobiotics but also by modulating host detoxification gene expression and immune-regulated defense pathways [164]. Endosymbionts such as *Wolbachia* and *Rickettsia* have been shown to alter insecticide susceptibility in a context-dependent manner, with effects that vary according to host species, symbiont strain, and the specific chemical exposure. These modulatory effects are primarily mediated through alterations in host metabolic processes, immune responses, and gene regulatory networks [165].

For example, *Wolbachia* enhances pesticide resistance in *T. urticae* by upregulating detoxification-related genes such as CYPs and GSTs, thereby increasing enzyme activity [166]. RNAi experiments confirmed that the genes *TuCYP392D2* and *TuGSTd05* are essential for this resistance phenotype. Notably, exposure to abamectin results in elevated *Wolbachia* levels, while the overall microbiome composition remains unchanged, highlighting the pivotal role of *Wolbachia* in augmenting detoxification and resistance to insecticides [166].

In *N. lugens*, *Wolbachia* enhances resistance to imidacloprid by promoting the expression of detoxification genes, including CYPs (e.g., *CYP6ER1*, *CYP6AY1*, *CYP4CE1*) and GSTs, potentially through activation of the CncC oxidative stress response pathway. This regulatory effect is temperature-sensitive: elevated temperatures reduce *Wolbachia* abundance, which in turn is associated with a decrease in detoxification gene expression and a corresponding decline in resistance to insecticides [167]. In contrast, an S-type strain of *Arsenophonus* has been shown to suppress host xenobiotic metabolism and reduce imidacloprid resistance in *N. lugens*, as evidenced by the downregulation of detoxification-related genes and altered metabolic profiles [168]. These findings demonstrate that the identity of the symbiont strain plays a crucial role in differentially modulating host detoxification capacity and insecticide susceptibility.

Further, the gut microbiota of honeybees, which is predominantly composed of *Lactobacillus*, exhibits temporal fluctuations during overwintering, with strain-specific variations that may influence immune function, metabolism, and overall health [169]. Microbial symbionts also contribute to toxin resistance by regulating cytochrome P450 gene expression in the midgut, thereby facilitating detoxification [170]. Collectively, these studies underscore the environmentally responsive nature of symbiont-mediated detoxification mechanisms and emphasize the vulnerability of these microbe-dependent resistance pathways to thermal stress.

In addition to influencing host gene expression, some microbiota confer resistance through direct metabolic detoxification. For example, *N. lugens* can acquire environmental *Serratia marcescens* capable of degrading buprofezin; gain or loss of this symbiont alters resistance levels, confirming its functional role in xenobiotic metabolism [171]. In *Bactrocera dorsalis*, *Citrobacter* sp. (CF-BD) carries phosphatase hydrolase genes that enhance survival under trichlorfon exposure [158]. Symbionts can also contribute to detoxification via distinct enzymatic pathways, as demonstrated in *D. melanogaster*, where gut microbiota mediate nitro-reductive imidacloprid metabolism in parallel with the host’s oxidative CYP6G1 pathway [172]. Similar dual-function mechanisms have been reported in other species. In *A. gossypii*, the gut symbiont *Sphingomonas* mediates imidacloprid resistance through both chemical degradation and upregulation of host cytochrome P450 genes, indicating a combined direct and indirect mode of action [173]. Likewise, in the bean bug *Riptortus pedestris*, *Burkholderia* symbionts degrade organophosphates such as fenitrothion while simultaneously inducing host gene expression changes that enhance resistance traits [161]. These findings highlight the complex and multifaceted contributions of insect-associated microbes to detoxification and the evolution of resistance.

Beyond direct enzymatic activity and gene regulation, symbiotic bacteria also influence resistance to insecticides by modulating host immune pathways. In *Lymantria dispar* larvae, shifts in the Gram-negative/Gram-positive composition of gut microbiota alter expression of Toll and IMD signaling components [174]. These immune pathways intersect with oxidative stress regulators such as Nrf2, which play a key role in the induction of detoxification enzymes. As a result, microbial regulation of host immunity represents an additional, indirect mechanism through which the microbiota can shape xenobiotic tolerance [175]. Table 3 summarizes key cases of microbiome-mediated insecticide resistance in insects, highlighting mechanisms like direct metabolism, gene regulation, and immune modulation.

### 3.4. Horizontal Gene Transfer (HGT) as a Driver of Novel Resistance Mechanisms

Emerging evidence indicates that horizontal gene transfer (HGT)—particularly from microbial partners such as bacteria or bacteriophages into insect genomes—can confer novel detoxification or immune effector capabilities. In *M. persicae*, fungal-origin horizontally transferred genes (HTGs) associated with cyanate detoxification have been shown to reduce reproduction when silenced, underscoring their functional role in managing toxic stress [176]. In gall midges (*Contarinia nasturtii*) and other Cecidomyiidae, microbial toxin genes including *cdtB*, *aip56*, *rhs*, and *sltxB* have been horizontally acquired and retain conserved catalytic residues, suggesting a contribution to insect adaptation through modulation of host interactions or immune responses [177]. Similarly, in the whitefly *B. tabaci*, a gene encoding a plant-derived detoxification enzyme appears to have been acquired via virus-mediated inter-kingdom transfer from host plants, potentially influencing both host-plant interactions and insecticide metabolism [178]. In Lepidoptera, the majority of identified HTGs are implicated in physiological roles such as nutritional metabolism and detoxification [179].

In *R. pedestris*, gut symbionts degrade the organophosphate insecticide fenitrothion into a non-toxic compound via a horizontally acquired enzyme. This bactericidal byproduct is subsequently excreted by the host, constituting a coordinated “host-symbiont detoxification relay” that maintains symbiosis while conferring effective resistance to insecticides. This mechanism parallels host-encoded detoxification pathways observed in other insects, highlighting the adaptive potential of microbial symbiosis and horizontal gene transfer in overcoming chemical stress [161].

Although direct evidence for HGT-derived detoxification genes in major agricultural pests remains limited, existing precedents from other insect species suggest these mechanisms may be more widespread and warrant further exploration. Recent reviews emphasize that bacterial symbionts and their associated bacteriophage communities represent a rich reservoir of genetic material capable of horizontal transfer, likely contributing to genome innovation in insects, particularly within ecological niches subjected to insecticide pressure [180,181]. These findings highlight the need to explore ecological, behavioral, and environmental factors in future resistance research.

### 3.5. Future Directions

Emerging evidence points toward even more complex and integrative resistance mechanisms that include: (1) Microbial symbioses may confer metabolic capabilities or modulate host immunity in ways that enhance insecticide tolerance [182]. (2) Behavioral adaptations, including altered movement and feeding patterns, can reduce exposure to treated surfaces [183]. (3) Climate change may accelerate resistance evolution not only through altered selection pressures and life histories [184], but also via temperature-dependent modulation of detoxification pathways—where elevated temperatures enhance cytochrome P450 activity and expression in pests like *Ae*. *aegypti* and *B*. *tabaci*, thereby increasing metabolic resistance to pyrethroids and neonicotinoids [184]. Together, these discoveries expand the resistance paradigm and highlight the adaptive plasticity of pest species subject to chemical control.

## 4. Cross-Resistance and Multiple Resistance in Insect Pests—Mechanisms, Environmental Factors, and Management Strategies

### 4.1. Resistance Types and Their Management Implications

#### 4.1.1. Cross-Resistance: One Mechanism, Many Failures

Cross-resistance poses a significant challenge to insecticide rotation strategies. This form of resistance often emerges silently in field populations under diverse chemical exposure, highlighting the value of early diagnostic screening for broad-spectrum enzymatic activity [185,186]. Notably, cross-resistance is often concentrated in polyphagous pests exposed to diverse insecticide chemistries [187,188], such as *Spodoptera* spp. and *P. xylostella*, due to their broad host range and frequent insecticide encounters. It occurs when a single genetic or biochemical mechanism confers resistance to multiple insecticides, sometimes spanning chemically unrelated classes. The most common drivers are detoxification enzymes with broad substrate specificity, particularly P450s. For instance, in *S. exigua*, the enzyme CYP321A8 metabolizes pyrethroids (cypermethrin and deltamethrin) and organophosphates (chlorpyrifos). This multifunctionality is mediated by two mechanisms: transcription factor overexpression and promoter mutations creating a new cis-regulatory element for orphan nuclear receptor binding [189]. The P450 gene *CYP321A8* also contributes to insecticide cross-resistance in field populations of *S. frugiperda* [190]. Further, Zuo et al. [191] studied the roles of different resistance mechanisms in *S*. *exigua* using nearly isogenic lines. They found that the L1014F mutation in the VGSC led to a resistance factor of 6.2, while the overproduction of the cytochrome P450 enzyme CYP9A9 resulted in a much higher resistance factor of 79. When both mechanisms were present, the resistance factor increased to 631 [191]. Similarly, P450s in *S*. *litura* drive indoxacarb resistance. Silencing *CYP339A1*, *CYP340G2*, and *CYP321A19* increased sensitivity, while transgenic overexpression enhanced resistance in *Drosophila*. These P450s may also mediate cross-resistance to chlorantraniliprole, λ-cyhalothrin, and imidacloprid [192].

Not all mechanisms of resistance rely on metabolism. For instance, chlorfenapyr resistance in *P. xylostella* is primarily governed by one major gene or a few tightly linked loci, with autosomal and incompletely dominant inheritance [193]. This resistance exhibited minimal involvement of detoxification enzymes and showed no significant cross-resistance to other insecticides, which suggests a distinct mechanism, potentially involving mitochondrial disruption or non-specific energy metabolism effects rather than typical target-site or metabolic alterations.

Environmental stressors complicate resistance dynamics. Exposure to heavy metals like cadmium (Cd) can trigger overexpression of detoxification genes through activation of the ROS/CnCC signaling pathway, enhancing P450-mediated detoxification and contributing to cross-resistance in Cd-exposed insects [194]. This phenomenon, known as xenobiotic preconditioning, can predispose pest populations to insecticide cross-resistance even without direct exposure to insecticides [195], which illustrates how anthropogenic pollution can inadvertently amplify resistance risks. In some pest species, such as *B. tabaci*, both cross-resistance and multiple resistance coexist, with overlapping metabolic and target-site mechanisms evolving under sustained and diverse chemical pressures [196].

#### 4.1.2. Multiple Resistance: Accumulation of Independent Mechanisms

Multiple resistance arises when pest populations independently acquire mechanisms that impart resistance to several insecticide classes, resulting in a polygenic and often synergistic defense system [197,198]. Such resistance profiles are especially concerning when different mechanisms converge in a single population, reducing the utility of rotation or combination treatments. Unlike cross-resistance, which is typically mediated by a single mechanism (e.g., detoxification enzyme or target-site mutation) conferring resistance to structurally related compounds, multiple resistance involves distinct mechanisms selected by sequential or overlapping exposure to different chemistries. Multiple resistance is frequently observed in pests subjected to intensive insecticide regimes in monoculture cropping systems, such as *Chilo suppressalis* and *S. frugiperda*, where diverse insecticide classes are applied over multiple generations, driving accumulation of resistance traits [197,199]. For example, *C. suppressalis* has evolved resistance to diamides through multiple mutations in the ryanodine receptor gene (e.g., *I4758M*, *Y4667C*, *Y4667D*), with combinations of these mutations contributing additively to high levels of tetraniliprole resistance [200]. Tetraniliprole is a pyrazole carboxamide insecticide, which has the structure of cyantraniliprole in which the bromine atom has been replaced by a [5-(trifluoromethyl)-2H-tetrazol-2-yl]methyl group. It has a role as a ryanodine receptor agonist [201]. In *S. frugiperda*, resistance-associated mutations have been identified across multiple target-site genes—*ace-1*, *CHSA*, *GluCl*, and *RyR*—indicating the potential for multiple resistance to organophosphates, chitin synthesis inhibitors, ivermectins, and diamides [56,186].

Regulatory mechanisms also play a role. In *N. lugens*, cis-regulatory variation upstream of *CYP6CS1* enhances P450 expression, conferring resistance to pymetrozine and moderate cross-resistance to other insecticides [202]. Additionally, resistance evolution often entails fitness trade-offs. In *A. gossypii*, sulfoxaflor resistance declined after selection pressure was removed, but reduced phloem-feeding efficiency and lower relative fitness persisted, illustrating the lingering costs of resistance, even after phenotypic reversion [203].

#### 4.1.3. Strategic Implications: Toward Mechanism-Informed IRM

Addressing these resistance patterns demands more than chemical rotation—it requires tactical integration of molecular diagnostics and ecological insights. The intertwined challenges of cross-resistance and multiple resistance call for a fundamental shift in the management of resistance to insecticides (IRM). Traditional rotation strategies, once the cornerstone of resistance delay, often fall short when resistance alleles are already widespread or when cross-resistance networks link multiple insecticides. *T*. *absoluta* populations resistant to tetraniliprole (a diamide) also show cross-resistance to chlorantraniliprole and flubendiamide, but remain susceptible to other insecticides like emamectin benzoate, broflanilide, spinosad, metaflumizone, and indoxacarb [204]. This pattern restricts the effectiveness of simple rotation schemes. These examples demonstrate how detailed resistance profiling—identifying specific cross-resistance patterns and independent resistance mechanisms—can guide more precise insecticide selections that minimize efficacy loss and delay resistance evolution. Rotations effectively delay resistance only when insecticides have non-overlapping modes of action and are applied to mostly susceptible pest populations—conditions that are rarely found in the field [205].

To overcome these challenges, IRM must incorporate integrated, mechanism-informed strategies such as (1) *high-throughput diagnostics tools*: including qPCR assays for detoxification gene expression and allele-specific PCR for key resistance mutations (e.g., *kdr*, *RyR*), enabling real-time resistance monitoring and targeted interventions [206]. (2) *Synergist-enhanced formulations*: Combining insecticides with metabolic enzyme inhibitors, such as PBO, can restore efficacy by blocking key resistance pathways. Novel approaches like RNAi offer promising routes to bypass known resistance mechanisms altogether [207]. (3) *Ecological management*: Mitigating environmental stressors, such as heavy metal contamination that can pre-activate detoxification pathways, reduces unintended induction of resistance. Concurrently, integrating biological control agents (e.g., *Trichogramma* spp.) diversifies pest mortality sources and disrupts selection for resistance [208,209]. (4) *Exploiting fitness costs*: Where resistance imposes biological trade-offs, temporarily withdrawing or rotating insecticides can enable susceptible genotypes to rebound, slowing resistance spread [203].

Ultimately, sustainable IRM demands abandoning one-size-fits-all prescriptions in favor of dynamic, data-driven strategies that leverage detailed biochemical, ecological, and genomic insights. Such integrated approaches will better guide insecticide deployment in both space and time, preserving control efficacy and agricultural productivity [205,210].

### 4.2. Resistance Management: A Multitactic Framework for Insecticide Sustainability

**Figure 2:**
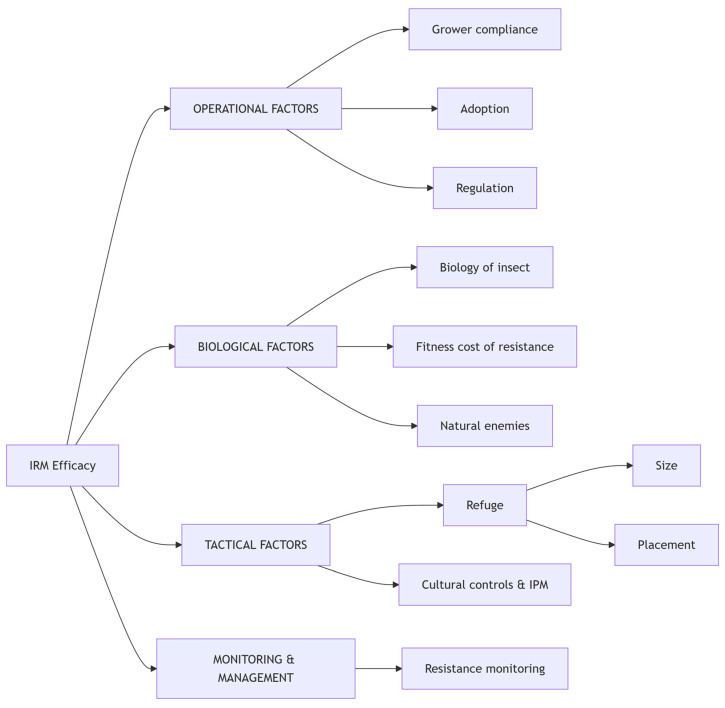
Conceptual framework illustrating the multifactorial influences on the efficacy of Insect Resistance Management (IRM) strategies. The diagram organizes contributing factors into four major domains: *Operational Factors*, which include grower compliance, adoption rates, and regulatory oversight; *Biological Factors*, encompassing insect biology, fitness costs linked to resistance alleles, and interactions with natural enemies; *Tactical Factors*, covering specific IRM implementation strategies such as refuge size and placement, integration with cultural control methods, and broader IPM programs; and *Monitoring & Management*, emphasizing the critical role of continuous resistance surveillance. The arrows indicate the hierarchical and interconnected relationships among these elements, demonstrating how biological constraints, management practices, and stakeholder behavior collectively influence IRM outcomes.

The multifactorial nature of factors influencing IRM efficacy is summarized in Figure 2, which highlights the interplay between operational, biological, tactical, and monitoring components essential for successful IRM. Resistance to insecticides poses an escalating threat across agricultural, public health, and urban sectors, demanding comprehensive, science-based management. Mode-of-Action (MoA) rotation, a cornerstone strategy, reduces selection pressure by alternating chemistries with distinct biochemical targets. The IRAC classification system guides evidence-based rotations, particularly in agriculture, where chemical diversity and pyramided *Bt* crops (e.g., cotton and maize) delay resistance development [211,212,213]. For instance, Australia’s cotton industry implemented mandatory IRAC MoA rotation since the 1990s, restricting consecutive applications of any insecticide class to a single generation of *Helicoverpa* pests. This strategy—combined with refuge crops and threshold-based spraying—reduced pyrethroid resistance frequencies from >50% to <10% within 5 years while extending the efficacy of newer chemistries like diamides [211]. However, cross-resistance complicates this approach. Metabolic mechanisms such as CYPs upregulation and target-site mutations like kdr can confer resistance across multiple insecticide classes [131,185]. Therefore, effective IRM requires molecular diagnostics and surveillance data to inform timely and tactical shifts in insecticide use.

Complementary strategies enhance IRM resilience. Insecticide mixtures and synergists, which combine full doses of active ingredients with independent MoAs, significantly reduce the risk of pests developing dual resistance [214]. Innovations such as next-generation long-lasting insecticidal nets (LLINs) that combine pyrethroids with synergists like PBO or novel insecticides, such as chlorfenapyr, exemplify this strategy in vector control [215,216]. Refugia strategies, especially structured non-*Bt* plantings in transgenic crop systems, preserve susceptible genotypes and dilute resistance alleles through interbreeding [217,218]. Although refugia are less feasible in public health and urban contexts, the conservation of natural enemies offers partial mitigation by maintaining ecological balance [219,220,221].

The successful implementation of the management of resistance to insecticides depends on several pillars. Surveillance-driven decision thresholds, using bioassays [222] and molecular tools, such as allele frequency tracking, trigger interventions before resistance becomes widespread [223,224,225,226,227]. Diversified tactics beyond chemicals, including biological control agents like entomopathogens, RNAi, gene drives, and ecological methods such as attract-and-kill, reduce reliance on insecticides and delay resistance evolution [228,229,230,231]. Finally, robust policy frameworks like the WHO Global Plan for Integrated Resistance Management (GPIRM) and IRAC guidelines provide essential structures for coordinated action, while stakeholder education and capacity building ensure adaptive and effective execution [232,233,234]. In summary, the proactive integration of these elements across both institutional and ecological contexts is vital to sustaining insecticide efficacy and protecting human health and agricultural productivity into the future.

### 4.3. Divergent Strategies for RNAi Resistance to Insecticides Management, a Comparative Framework for Transgenic and Sprayable Applications

#### 4.3.1. Distinct Exposure Dynamics Drive Divergent IRM Needs

The contrasting exposure profiles of RNAi-based insecticides, transgenic versus sprayable, fundamentally shape their IRM strategies. Transgenic crops such as SmartStax PRO^®^ maize, which express *DvSnf7* dsRNA, expose target pests like western corn rootworm to season-long, continuous selection pressure. This persistent exposure creates a high-risk environment for the evolution of resistance across multiple overlapping generations in the field [207,235]. In contrast, sprayable dsRNA products like Calantha^®^ (Ledprona^®^), which target the Colorado potato beetle, were developed specifically for pest management and are applied intermittently [236]. The rapid environmental degradation of dsRNA [237] limits exposure to short, defined treatment windows, ideally targeting a single pest generation—often the early larval stage. These fundamental differences in exposure duration and intensity demand distinct IRM designs: long-term containment for transgenic traits versus generation-specific rotation for foliar sprays.

#### 4.3.2. Tailored IRM Strategies Reflect Product Format and Biology

For transgenic RNAi insecticides regulated as Plant-Incorporated Protectants (PIPs), IRM strategies focus on pyramiding and refuge requirements. Products like SmartStax PRO^®^ combine dsRNA with *Bt* proteins (e.g., Cry3Bb1 and Gpp34Aa/Tpp35Aa) to deliver multiple, non-cross-resistant modes of action within a single crop. This approach ensures that even if resistance arises to one component, the other maintains efficacy [235,238]. To further delay resistance, a structured refuge of 5% non-transgenic seed is included in seed mixtures. This practice promotes mating between susceptible and potentially resistant individuals, leveraging the recessive nature of dsRNA resistance inheritance to reduce the frequency of homozygous-resistant offspring [239].

Management of resistance of insects to management for sprayable dsRNA, categorized under IRAC Mode of Action Group 35, relies on temporal separation of exposures via mode-of-action (MoA) rotation. Products are applied within defined “treatment windows,” typically limited to one application per pest generation, followed by rotation to a distinct MoA group (e.g., IRAC Groups 4 or 28) for the next generation or application [233]. This strategy takes advantage of the lack of cross-resistance between dsRNA and small-molecule insecticides, reducing selection pressure continuity and preserving dsRNA efficacy [240].

#### 4.3.3. Mechanisms of Resistance Inform Risk and Regulatory Design

Both strategies are informed by a shared mechanistic understanding of dsRNA resistance in coleopteran pests. The predominant mechanism, impaired cellular uptake of dsRNA, exhibits autosomal recessive inheritance and confers cross-resistance among RNAi targets, but not to *Bt* toxins or conventional chemical insecticides [239,240,241]. Laboratory selection studies have demonstrated that resistance can evolve rapidly, within as few as 7–11 generations, and resistant populations can suffer little due to a lack of fitness cost in the absence of selection [242]. These findings reinforce the necessity of early and robust implementation of IRM to prevent widespread resistance. Each approach aligns with regulatory frameworks designed for each system of delivery. Transgenic RNAi traits must adhere to EPA guidelines that emphasize high-dose expression, pyramiding of non-cross-resistant traits, structured refuges, and resistance monitoring protocols [238,243,244]. Alternatively, Sprayable RNAi insecticides are seamlessly incorporated into global MoA-based IRM systems developed by the Resistance to Insecticides Action Committee (IRAC), including principles of rotation and generational treatment limits [245]. Going forward, harmonizing these IRM strategies across international jurisdictions will be critical for supporting the long-term sustainability of RNAi technologies in IPM programs [207].

## 5. Case Studies

The mosquito *Ae*. *aegypti* and the fall armyworm *S*. *frugiperda* were selected as representative case studies due to their global economic and public health significance, their rapid evolution of resistance, and the extensive research characterizing their underlying mechanisms across diverse ecological and regulatory contexts [246,247].

### 5.1. Mechanisms of Resistance to Insecticides for the Mosquito, Aedes aegypti

The extensive and prolonged application of insecticides has exerted intense evolutionary pressure on mosquito populations, particularly *Ae. aegypti*, fostering the emergence of resistance (Table 4). Resistance to insecticides is widely considered a pre-adaptive process, whereby alleles conferring survival advantages may be present at low frequencies within populations prior to exposure to selective agents [248]. However, resistance can also arise from de novo mutations following insecticide application. Individuals harboring these alleles exhibit increased survival and reproductive success, leading to a rise in resistance allele frequencies. Two principal mechanisms underlie this resistance: alterations at the insecticide target site and enhanced metabolic detoxification [124].

#### 5.1.1. Target Site Resistance

Target site resistance arises from nucleotide substitutions that lead to changes in amino acids in critical proteins, which reduce the binding affinities of insecticides. Notably, mutations in the AChE genes, *Ace1* and *Ace2*, have been implicated in resistance to organophosphates in species such as *An. gambiae*, *Cx. pipiens*, and *Culex tritaeniorhynchus* [249,250,251]. However, conclusive evidence of this mechanism in *Ae. aegypti* remains to be done.

**Figure 3:**
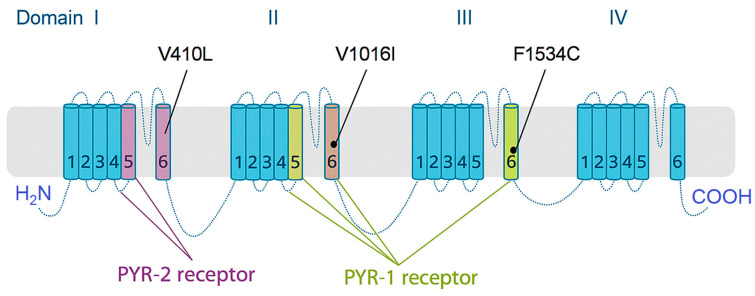
Schematic representation of three key amino acid substitutions—V410L, V1016I, and F1534C—in the *Aedes aegypti* voltage-gated sodium channel (VGSC). These mutations are located in domain I segment 6 (IS6), domain II segment 6 (IIS6), and domain III segment 6 (IIIS6), respectively. The domain segments and connecting helices contributing to pyrethroid receptor sites 1 (green) and 2 (purple) are also highlighted. These structural changes are associated with knockdown resistance (kdr) to pyrethroids. Reproduced from [258]. Copyright 2018, Nature Portfolio.

Resistance to pyrethroids, often termed “knockdown resistance” (*kdr*), involves point mutations in the VGSC (Figure 3), a primary target of these insecticides. Such mutations have been identified across a broad spectrum of arthropods, including both pest and vector species [64]. These mutations may influence channel gating dynamics, affecting insecticide binding efficacy. Eleven VGSC mutations associated with pyrethroid resistance have been identified in *Ae. aegypti*.

##### V1016I Mutation

The V1016I mutation, identified by [252], entails a G → A substitution at codon 1016 in domain II, segment 6 (IIS6) of the VGSC. This single-nucleotide polymorphism results in a valine-to-isoleucine amino acid change. While this mutation alone may not significantly alter insecticide susceptibility, it often co-occurs with F1534C, and their combination appears to synergistically impair insecticide binding [64,253].

##### F1534C Mutation

F1534C, first observed in populations from the Grand Cayman Islands and Thailand [254,255], results from a T → G transversion at the second position of codon 1534 in domain III, segment 6 (IIIS6). This leads to a substitution of phenylalanine with cysteine. Functional studies have demonstrated that F1534C confers resistance primarily to type I pyrethroids, with limited efficacy against type II compounds (Figure 3) [256].

##### V410L Mutation

The V410L mutation (Figure 3), first identified in 2017 in a Brazilian laboratory strain of *Ae. aegypti* [257], results from a G → T transversion at the first nucleotide of codon 410 in domain I, segment 6 (IS6), leading to a valine-to-leucine amino acid substitution. Although initially absent from wild populations, its frequency has increased substantially over time. In Tapachula, Mexico, by 2016, heterozygosity at the 410 locus had increased to 64% [258]. This trend paralleled increases in the frequencies of V1016I and F1534C, and a strong genotype–phenotype correlation was established for resistance to both type I (e.g., permethrin) and type II (e.g., deltamethrin) pyrethroids [258].

#### 5.1.2. Metabolic Resistance

Enzymatic detoxification represents another major mechanism whereby *Ae. aegypti* mitigates insecticidal toxicity. Elevated levels of CES activity have been associated with phenotypes resistant to insecticides [259]. Overexpression of several detoxifying enzyme families, including CYPs, carboxyl/cholinesterases (CCEs), and GSTs, has been documented in resistant populations of mosquitoes [246,260,261]. These enzymatic systems often work in concert, with CCEs initiating pyrethroid hydrolysis, followed by further metabolism via CYPs [262]. Such cross-functional enzymatic activity can result in broad-spectrum resistance, independent of the insecticide’s target site [131].

#### 5.1.3. Resistance to Insecticides and Associated Fitness Costs

The evolutionary dynamics of resistance to insecticides are intertwined with vector fitness and epidemiological outcomes. While resistance mechanisms can impose physiological trade-offs that affect vectorial capacity and may lead to reversion toward susceptibility in insecticide-free environments, this is not universally the case [263]. Some resistance alleles, such as the *Ae. aegypti* V1016I *kdr* mutation, exhibit low fitness costs and can persist in populations even in the absence of selection pressure [264]. Thus, the persistence or decline of resistance alleles depends on the balance between fitness costs and benefits in the specific ecological context [265,266].

Several longitudinal studies have explored the consequences of resistance to insecticides on fitness [267,268]. Evaluating fitness requires careful measurement of key life-history traits, including adult longevity, host-seeking behavior, reproductive success (e.g., fecundity and egg viability), and larval development [265,269,270,271]. However, these comparisons must be cautiously interpreted. These physiological costs can reduce a mosquito’s vectorial capacity under pesticide stress. They do so by shortening its lifespan or impairing its pathogen defense mechanisms [272,273,274]. Resistance phenotypes may result from multiple interacting mechanisms, and variations in environmental adaptation among field and laboratory strains can confound conclusions [269,275].

Further research is required to elucidate the extent and ecological significance of these trade-offs, particularly in multi-generational contexts and under varying environmental conditions. A deeper understanding of resistance-associated fitness costs holds twofold importance: it can help predict the reversibility of resistance post-intervention and inform vector control strategies by evaluating how these costs affect mosquito population dynamics and pathogen transmission. Importantly, regional differences in insecticide regulations and use patterns contribute to the selection of distinct resistance alleles and mechanisms in *Ae*. *aegypti* populations worldwide. For example, pyrethroid resistance is more prevalent in Latin America and Southeast Asia due to historical overuse, while organophosphate resistance remains significant in Africa [246,276,277,278]. These differences underscore the need for locally adapted vector control strategies.

### 5.2. Mechanisms of Resistance to Insecticides in Spodoptera frugiperda

The fall armyworm (*S. frugiperda*, (FAW)) exhibits multiple, often concurrent, resistance mechanisms that complicate efforts to control with insecticides (Table 4). These include metabolic detoxification, target-site mutations, reduced cuticular penetration, and behavioral avoidance. Metabolic resistance is primarily mediated by elevated activity of detoxification enzymes, such as CYPs, GSTs, and CESs. For instance, overexpression of CYP genes, including *CYP6AE43*, *CYP321A8*, and *CYP321A9*, has been linked to enhanced degradation of diamide insecticides such as chlorantraniliprole [70].

Target-site resistance arises from mutations that alter the binding affinity of insecticides. The I4790K mutation in the RyR reduces the efficacy of diamides [186], while point mutations such as F290V and A201S in AChE confer resistance to organophosphates and carbamates [56,224,279,280]. Additional mechanisms, including decreased cuticular penetration and avoidance behaviors of larvae, reduce insecticide exposure [161]. These mechanisms frequently co-occur, resulting in widespread multi-resistance to insecticides in FAW populations globally [281,282].

#### 5.2.1. Global Distribution and Severity of Resistance

Resistance levels vary markedly across regions, reflecting local insecticide use patterns. In China, field populations of *S*. *frugiperda* have developed moderate to high resistance to indoxacarb (resistance ratio [RR] = 9.23–45.53×) and spinetoram (RR = 4.32–18.05×), with incipient signs of reduced susceptibility to chlorantraniliprole [159]. Egyptian *S*. *frugiperda* populations exhibit moderate resistance to cypermethrin (RR = 3.65–5.75), with metabolic detoxification, particularly GST activity, contributing to resistance. Emamectin benzoate remains highly efficacious, while synergists enhance spinosad toxicity, supporting an enzyme-mediated resistance mechanism [283].

In Brazil, *S. frugiperda* populations have evolved high resistance levels to diamide insecticides—including flubendiamide, chlorantraniliprole, cyantraniliprole, and tetraniliprole—primarily mediated by target-site mutations in the RyR, notably I4790M and I4790K substitutions [284]. Genetic analyses indicate resistance is inherited as an autosomal, incompletely recessive, monogenic trait. Robust cross-resistance among diamides has been documented. Although the overall frequency of these resistance-associated alleles remains low in many regions, locally elevated frequencies underscore the urgent need for integrated resistance management strategies to sustain diamide efficacy in fall armyworm control [186].

Genetic analysis of invasive *S. frugiperda* populations in India revealed low nucleotide diversity and close genetic affinity to African populations, indicating recent common origins. Upregulation of detoxification genes (CYPs, GSTs) underlies observed resistance to insecticides, with resistance ratios significantly correlated to specific loci (e.g., GST1950, CYP9131, CYP9360), highlighting the contribution of heritable metabolic mechanisms to resistance development [285,286].

In Europe, invasive *S. frugiperda* populations—such as those in Greece—show alarmingly high frequencies (up to 80.9%) of ABCC2 gene mutations, molecular markers associated with resistance to *Bt* Cry toxins, emphasizing an urgent need for rigorous resistance monitoring [287].

The diversity of regulatory frameworks and insecticide usage across continents has resulted in geographically distinct resistance profiles in *S. frugiperda*. For example, the rapid emergence of diamide resistance in Brazil contrasts with the slower development of resistance in parts of Africa, where *Bt* crops and conventional chemistries are deployed differently [281,284].

#### 5.2.2. Integrated Resistance Management Strategies

The effective management of resistance in *S. frugiperda* necessitates an integrated and multifaceted approach. Strategic rotation of insecticides with distinct modes of action—such as alternating diamides (Group 28) with spinosyns (Group 5)—is imperative to delay the onset of resistance [288,289]. Furthermore, the application of synergists, notably diethyl maleate (DEM), has been shown to significantly enhance insecticidal efficacy, augmenting spinosad toxicity by up to eightfold in resistant populations [283].

Biological control constitutes a sustainable and ecologically sound strategy for managing *S. frugiperda*; however, its global implementation remains constrained and methodologically fragmented. Although over 500 natural enemies have been documented—including approximately 304 parasitoids, 215 predators, and 46 entomopathogens—only approximately 40% have been subjected to laboratory or field evaluations, with fewer undergoing rigorous population-level efficacy assessments [290]. Notably, isolates of *Beauveria bassiana* have exhibited considerable potential, eliciting up to 87% egg mortality and 71–93% cumulative larval mortality within 14 days, in addition to suppressing feeding by second-instar larvae by 69–78% within 48 h [291]. Despite these promising results, geographic and taxonomic biases, insufficient bio-inventories, and the absence of standardized efficacy metrics impede the extrapolation of experimental findings to broad-scale field applications.

Unlike chemical insecticides, biological control agents seldom achieve complete eradication; instead, they promote sustained population suppression within ecological thresholds, preserving natural enemy communities and reducing the risk of pest resurgence [292]. Complementary cultural practices, such as push-pull strategies and optimized planting schedules, have been demonstrated to substantially enhance early-season pest suppression. For example, a climate-adapted push-pull system implemented in East Africa, which involves intercropping maize with drought-tolerant *Desmodium* species and deploying *Brachiaria* cv. Mulato II, as a border crop, has yielded significant reductions in pest infestation and crop damage, concurrently improving maize yields [293].

Ultimately, the successful reduction of insecticide overuse and the effective adoption of IPM frameworks are contingent upon comprehensive farmer education and active stakeholder engagement [294].

#### 5.2.3. Future Directions for Resistance Mitigation

Long-term control of FAW will depend on genomics-guided surveillance as well as other emerging technologies. Molecular diagnostics allow for real-time detection of resistance-associated alleles, such as RyR I4790K, in field populations [287]. New insecticides, including fluxametamide—a novel isoxazoline insecticide (Group 30)—exhibit low resistance potential, with a realized heritability (h^2^) of 0.084 and only a 2.63-fold increase in LC_50_ after 10 generations of selection. The selected strain showed no significant cross-resistance except a modest increase to emamectin benzoate and displayed fitness costs, including reduced reproduction and development (relative fitness = 0.353) [163].

Biotechnological advances such as RNAi targeting essential genes like chitin synthase and transgenic crops expressing multiple *Bt* toxins offer promising control strategies. However, their success hinges on proper refuge management to mitigate resistance evolution [186]. The establishment and maintenance of global resistance databases, such as the FAO’s FAW portal, are essential for harmonized data reporting and informed decision-making [8]. Finally, future investment in next-generation control tools—including nanoparticle-based formulations and mycoinsecticides—holds potential to enhance efficacy while minimizing environmental impact [295].

*Closing remarks*—The diverse resistance mechanisms and control challenges observed in *S*. *frugiperda* contrast with those in *Ae*. *aegypti*, reflecting their distinct life histories, ecologies, and insecticide selection pressures. Together, these two species illustrate divergent evolutionary trajectories and multifaceted resistance adaptations shaped by their biology and exposure histories. This comparison underscores the critical importance of tailoring IRM strategies to species-specific vulnerabilities and local chemical selection landscapes.

## 6. Strategies for Managing Resistance to Insecticides: Chemical, Biological, and Integrated Approaches

The escalating threat of resistance to insecticides in pest populations necessitates a paradigm shift in pest control strategies. Traditional reliance on chemical insecticides has proven insufficient and environmentally detrimental in the long term. Therefore, modern pest management emphasizes integrated approaches that combine chemical, biological, and ecological tactics to delay resistance evolution and minimize ecological impacts (Table 5).

### 6.1. Conventional Insecticides: Effectiveness and Limitations

Insecticides such as organophosphates, pyrethroids, and neonicotinoids remain essential in current pest management frameworks due to their broad-spectrum activity and rapid knockdown effects [125]. However, their efficacy is increasingly undermined by widespread resistance—driven by metabolic, target-site, penetration, behavioral, and emerging sequestration mechanisms—and by cross-resistance across chemical classes [296]. Operational factors such as poor spray coverage, mistimed application, and pest cryptic behavior further reduce control success [297]. Moreover, their environmental persistence and broad-spectrum toxicity lead to non-target impacts, ecosystem imbalance, pest resurgence and secondary outbreaks, soil and water contamination, and human health hazards from acute and chronic exposure [298]. For instance, neonicotinoids, despite their systemic properties and efficacy against sap-feeding pests, have been implicated in the disruption of pollinator navigation and colony collapse disorder [299].

Sulfoxaflor, a next-generation sulfoximine insecticide, targets sap-feeding pests resistant to neonicotinoids by acting as a nAChR agonist at a distinct receptor subtype. Initially deemed safer for pollinators, recent studies reveal sublethal effects on bees, including impaired foraging, disrupted gut microbiota, and reduced colony performance [300,301,302,303]. Thus, while chemical insecticides remain vital tools, they must be deployed judiciously within IRM frameworks to sustain efficacy and reduce ecological footprints.

These limitations underscore the growing role of IPM—including strategic rotation with biorational agents—to mitigate resistance development, reduce environmental burden, and sustain long-term effectiveness [304].

### 6.2. Next-Generation Resistance-Breaking Technologies

While IPM remains essential, truly durable solutions require disrupting resistance at its genetic roots. Such as (1) Nanocarrier-based delivery of dsRNA targeting long non-coding RNAs (e.g., lnc-*GSTu1*-AS) or resistance-related genes (e.g., *GSTu1*, *SAP2*, *NIOBP3*) enables effective RNAi-mediated suppression of detoxification pathways associated with resistance to insecticides. In *P. xylostella*, silencing lnc-*GSTu1*-AS destabilized GSTu1 transcripts and increased susceptibility to chlorantraniliprole [156]. Similarly, silencing *CYP6CY3* via nanocarrier-delivered dsRNA increased imidacloprid susceptibility in *A. gossypii* [305]. Broadly, nanoparticles—including liposomes, chitosan polymers, dendrimers, and lipid-protamine complexes—enhance delivery efficacy by protecting dsRNA from degradation, improving cellular uptake, and promoting endosomal escape, leading to stronger gene knockdown and pest control outcomes [306]. (2) CRISPR-Cas9 *allelic-drive* systems can replace resistant *kdr* mutations (e.g., V1016I) or amplified *P450* alleles with wild-type sequences, driving susceptibility through pest populations—a CRISPR-based allelic-drive in *D. melanogaster* reversed a resistant *kdr* mutation to the wild-type form in ~80–87% of the population within 8–10 generations [307]. (3) Machine learning models that integrate real-time genomic data (e.g., allele frequencies of resistance loci), insecticide usage patterns, and climate projections have demonstrated high predictive accuracy in identifying resistance hotspots in malaria vectors across Africa. Ensemble models, such as XGBoost, Random Forest, and Bayesian Generalized Additive Models, have been employed to predict insecticide resistance phenotypes, utilizing bioassay mortality data and a suite of covariates. These models have been validated using out-of-sample data, showing robust performance in forecasting resistance trends and informing targeted vector control strategies [308].

### 6.3. Biopesticides: Eco-Friendly Alternatives

Biopesticides derived from plants, microorganisms, and natural enemies offer eco-compatible alternatives to synthetic chemicals. These agents are often species-specific, biodegradable, and pose minimal risks to humans and non-target organisms [309,310]. Plant-based biopesticides—such as azadirachtin, pyrethrins, rotenone, ryania, nicotine, and sabadilla—exert neurotoxic, antifeedant, or growth-disrupting effects on pests. They are species-specific, biodegradable, and safer for non-target organisms, making them effective, eco-friendly alternatives in IPM [311]. Moreover, the widespread use of botanical products has reinvigorated traditional pest control knowledge and offers viable solutions for organic agriculture.

### 6.4. Biological Control: Natural Regulation of Pest Populations

Biological control employs natural enemies—predators, parasitoids, and entomopathogens—to suppress pest populations without chemical inputs. This approach is integral to IPM due to its high specificity, environmental safety, and sustainable efficacy [312,313]. Key entomopathogenic viruses include baculoviruses such as *H. armigera* nucleopolyhedrovirus (HaNPV) and *Spodoptera litura* nucleopolyhedrovirus (SliNPV), widely utilized across Asia and South America [314,315]. Entomopathogenic fungi, notably *B. bassiana* and *Metarhizium anisopliae*, infect over 700 pest species under optimal conditions [316]. Inoculative releases of parasitoids and predators in greenhouse and field environments provide long-term pest suppression [317]. Integrating biological control with biopesticides and selective chemical applications enhances pest management resilience while promoting biodiversity conservation [318].

## 7. Conclusions

The relentless application of insecticides has shaped a powerful evolutionary force, enabling insect pests to rapidly adapt through genetic and biochemical innovations. Resistance to insecticides evolves through two principal pathways: (1) target-site modifications, which hinder the binding of insecticides to their molecular receptors (e.g., nAChRs, AChE, VGSCs, GABA receptors), and (2) metabolic detoxification mechanisms, where the upregulation or amplification of key enzyme systems (esterases, CYPs, GSTs) neutralizes toxic compounds. These resistance strategies are often polygenic, heritable, and strongly influenced by selection pressure from the overuse or misuse of insecticides. This evolutionary pressure has led to a rich array of resistance mechanisms—behavioral shifts, thicker cuticles, mutated target sites, and enhanced detoxification pathways. While chemical pesticides remain indispensable tools for pest control, their diminishing efficacy, ecotoxicological consequences, and non-target impacts underscore the urgent need for integrated, sustainable alternatives. Recent studies confirm that resistance continues to escalate, with new alleles and cross-resistances appearing each year. For example, pyrethroid resistance in mosquitoes has intensified to the point that only new-generation interventions remain effective. Resistance management strategies offer tools to sustain insecticide utility. Integrated approaches, such as combining chemical and non-chemical tactics, rotating and mixing insecticides by mode of action, and conserving refugia, have been repeatedly shown to delay resistance emergence. Ongoing monitoring and adaptive management are key: resistance patterns vary over time and space, so tactics must be adjusted accordingly. Cross-sector learning is also valuable; for instance, the success of IPM in crops can inform vector control, and lessons from mosquito net rotations can inspire better practices in agricultural irrigation. As the post-insecticide era demands a fundamental reimagining of pest control, from reactive chemistry to proactive genetic disruption, leveraging evolutionary principles such as trade-offs, epistasis, and gene drive dynamics can help us outpace adaptation. Priority actions include establishing global consortia for real-time resistance allele tracking, fast-tracking regulatory processes for RNAi and nanotech tools, and developing open-source AI platforms to model resistance evolution. These transformative, ecology-guided strategies will be essential in securing food systems and public health, ensuring that insecticides remain effective for the foreseeable future.

## Figures and Tables

**Table 1:** Major mechanisms of resistance of insects to insecticides.

Mechanism	Description	Molecular/Behavioral Basis & Key Examples	Level & Pattern of Resistance	References
**Behavioral resistance**	Modified behaviors that reduce contact with treated surfaces.	- *Anopheles arabiensis*: Shift from indoor to outdoor resting - *Leptinotarsa decemlineata* (Colorado potato beetle): Avoidance of treated foliage - *Plutella xylostella* (Diamondback moth): Reduced oviposition on sprayed crops	Low–moderate (2–10-fold); delays physiological resistance; compromises contact-dependent interventions	[19,20,21]
**Penetration resistance**	Thickened or altered cuticle reduces insecticide uptake.	- *Aedes aegypti*, *Culex quinquefasciatus*: Epicuticular thickening via hydrocarbon remodeling - *Helicoverpa armigera*: Reduced deltamethrin penetration through enhanced cuticular esterases	Moderate (<5-fold); offers cross-class protection; synergizes with other mechanisms	[22,23]
**Target-site insensitivity**	Mutations at insecticide binding sites reduce compound efficacy.	- *kdr* (L1014F/Vssc): Pyrethroid/DDT resistance in *Anopheles gambiae* - *Ace-1*: OP/carbamate resistance via acetylcholinesterase (AChE) insensitivity - *Rdl* (A302S): Cyclodiene/fipronil resistance via GABA receptor changes	High (100–1000-fold); class-specific; causes rapid control failure; detectable via molecular diagnostics	[24,25,26]
**Metabolic resistance**	Increased detoxification via overexpressed or amplified enzymes.	- P450s: CYP6P9a/b overexpression in *An*. *funestus* (pyrethroid detoxification) - Carboxylesterases (CESs): *Coeae1f* amplification in *Ae*. *aegypti* (OP/pyrethroid hydrolysis) - GSTs: GSTe2 upregulation in *An*. *gambiae* (DDT dehydrochlorination)	High (10–500-fold); often polygenic; causes broad cross-resistance; undermines new insecticide chemistries	[27,28,29,30]

**Table 2:** Summary of nicotinic acetylcholine receptor (nAChR) mutations associated with resistance or susceptibility to neonicotinoid and spinosyn insecticides in major insect pests in the last decade.

Insect Species	nAChR Mutation(s)	Insecticide(s)	Resistance?	References
***Aedes aegypti* (yellow fever mosquito)**	No known nAChR target-site mutation	Neonicotinoids	No nAChR target-site mutations found (metabolic resistance via CYPs is highlighted elsewhere (Section 5.1.2))	[48]
	Frameshift mutation in α6 subunit (32-bp deletion)	Spinosad	Yes (320-fold resistance)	[49]
***Anopheles gambiae* (malaria mosquito)**	Multiple subunits. Reduced expression (β1, α3, α7)	Neonicotinoids (clothianidin, acetamiprid)	Partial resistance; 15–23-fold downregulation in field populations	[50]
***Drosophila melanogaster* (fruit fly)**	β1 subunit: R81T (engineered)	Neonicotinoids	Yes (Increased tolerance)—CRISPR/Cas9 genome editing; Fitness costs observed	[51]
	α6 subunit: Knockout	Spinosad	Yes (High resistance)—CRISPR/Cas9 deletion; No fitness deficits	[51]
***Ceratitis capitata* (medfly)**	α6 subunit: 3aQ68 * and K352 * (* premature stops)	Spinosyns (spinosad)	Yes—truncated α6 isoforms (stop codons at Q68 and K352) confer resistance	[52]
***Bemisia tabaci* (silverleaf whitefly)**	β1 subunit: A58T and R79E (target-site) [38]	Neonicotinoids (imidacloprid, thiamethoxam, etc.)	Yes—confers resistance [38]. (earlier studies suggested an absence of nAChR mutations [53])	[38,53]
	None identified	Spinosyns (spinosad)	No—no target-site mutations reported (resistance mainly metabolic)	[53]
***Tuta absoluta* (tomato borer)**	α6 subunit: G275E (target-site)	Spinosyns (spinosad, spinetoram)	Yes—high resistance via G275E	[54]
	None identified	Neonicotinoids	No—no nAChR target-site mutations reported in recent studies	
***Spodoptera exigua* (beet armyworm)**	α6 subunit: G275E (target-site)	Spinosyns (spinosad, spinetoram)	Yes—CRISPR knock-in G275E confers resistance	[55]
	None identified	Neonicotinoids	No—no target-site mutations reported	
***Spodoptera frugiperda* (fall armyworm)**	α6 subunit: G275E (low frequency)	Spinosyns	Potential transitional resistance—Amplicon sequencing (0.1–1% allele frequency)	[56]
***Plutella xylostella* (diamondback moth)**	α6 subunit: 3-amino-acid deletion (TM4)	Spinosyns (spinosad, spinetoram)	Yes—3-aa deletion (IIA) in nAChR α6 underlies ~940-fold resistance	[57]
	None identified	Neonicotinoids	No—no nAChR target-site mutation documented in recent literature	
***Frankliniella occidentalis* (western flower thrips)**	α6 subunit: G275E (target-site)	Spinosyns (spinosad)	Yes—G275E associated with spinosad resistance, but α6 knockout confers complete resistance to spinosad	[58]
***Thrips palmi* (melon thrips)**	α6 subunit: G275E (target-site)	Spinosyns (spinosad)	Yes—G275E confers spinosad resistance	[59]
***Myzus persicae* (green peach aphid)**	β1 subunit: R81T (major), V101I	Neonicotinoids (imidacloprid)	Yes—R81T and V101I linked to imidacloprid resistance	[33]
***Aphis gossypii* (cotton aphid)**	β1 subunit: R81T	Neonicotinoids (imidacloprid)	Yes—R81T confers neonicotinoid resistance	[60]
***Nilaparvata lugens* (brown planthopper)**	Nlα2 subunit	Neonicotinoids (imidacloprid, dinotefuran)	Yes—CRISPR/Cas9 knockout. Nlα2 knockout confers cross-resistance to neonicotinoids	[61]

Key: “Yes” indicates nAChR target-site resistance documented; “No” indicates no target-site mutation reported.

**Table 3:** Microbiome-mediated insecticide resistance mechanisms across insects.

Mechanism	Insect Host	Symbiont(s)	Pesticide(s)	Evidence Type	Notes	References
Direct metabolism	*Bactrocera dorsalis*	*Citrobacter* sp. (CF-BD)	Trichlorfon	Genomics + Metabolomics	Degrades trichlorfon into chloral hydrate and dimethyl phosphite; enhances host survival	[158]
Direct metabolism	*Riptortus pedestris*	*Burkholderia*	Fenitrothion	Bioassays + Functional Enzyme Tests	Soil-acquired strains detoxify fenitrothion; induces resistance and modulates host gene expression	[161]
Direct metabolism	*Nilaparvata lugens*	*Serratia marcescens*	Buprofezin	Gain/loss of symbiont	Acquisition alters resistance; symbiont breaks down pesticide	[171]
Direct metabolism	*Drosophila melanogaster*	Mixed gut microbiota	Imidacloprid	Metabolic comparisons	Symbiont-mediated nitro-reduction complements host oxidative CYP6G1 pathway	[172]
Gene regulation	*Nilaparvata lugens*	*Wolbachia*, *Arsenophonus*	Imidacloprid	Gene expression profiling	*Wolbachia* upregulates CYPs and GSTs; *Arsenophonus* suppresses detox genes; response is strain-specific	[167,168]
Gene regulation	*Apis mellifera*	Gut bacteria (e.g., *Pantoea*, *Enterobacter*)	Clothianidin	Transcriptomics + Probiotic Rescue	Disrupted microbiota leads to P450 gene suppression; reintroduction restores detox response	[170]
Gene regulation + Enzyme activity	*Tetranychus urticae*	*Wolbachia*	Abamectin, Pyridaben, Cyflumetofen	RNAi, qPCR, Enzyme Assays	Upregulates TuCYP392D2 and TuGSTd05; increases GST activity; abamectin increases *Wolbachia* abundance	[166]
Direct + Indirect (dual)	*Aphis gossypii*	*Sphingomonas*	Imidacloprid	Dual-mode functional profiling	Chemical degradation and host P450 upregulation demonstrated	[173]
Immune modulation	*Lymantria dispar*	Mixed gut microbiota	Pyrethroids	Immune gene profiling	Microbial shifts affect Toll/IMD signaling and Nrf2-mediated detox enzyme expression	[175]

**Table 4:** Comparative analysis of resistance to insecticides traits in *Aedes aegypti* and *Spodoptera frugiperda*, highlighting mechanisms, evolutionary dynamics, and control implications.

Trait	*Aedes aegypti*	*Spodoptera frugiperda*	Evolutionary Insight
**Primary mechanism**	Target-site (*kdr*) + P450s	Metabolic (P450s) + RyR mutation	Vector-herbivore divergence in adaptation
**Resistance spread**	~10–20 years regionally	<5 years globally	Trade-facilitated gene flow accelerates resistance
**Fitness cost**	Reduced fecundity, prolonged development	Minimal (behavioral compensation)	Metabolic flexibility buffers trade-offs
**Key vulnerability**	Sequestration (OBPs)	RNAi susceptibility	Taxon-specific weaknesses enable precision control

**Table 5:** Resistance to insecticide management (IRM) strategies.

Approach	Core Principle	Implementation Examples and Scientific Rationale
**MoA rotation**	Alternate MoA classes to disrupt resistance selection.	- Agriculture: Rotate neonicotinoids (MoA 4A) → spinosyns (MoA 5) → diamides (MoA 28) - Public health: IRS switch pyrethroids (MoA 3A) → carbamates (MoA 1A) annually
**Mixtures & synergists**	Combine compounds with distinct targets or inhibit detoxification.	- PBO-nets: Pyrethroid + piperonyl butoxide (P450 inhibitor) for *An*. *gambiae* control - AChE/GABA co-targeting: Neonicotinoid (MoA 4A) + fipronil (MoA 2B) tank mixes
**Refugia & IPM**	Maintain susceptible alleles; reduce pest density non-chemically.	- Structured refugia: ≥20% non-Bt maize adjacent to Cry1Ab fields - Cultural: Trap cropping (*Brassica* spp. for diamondback moth) + sanitation (container removal for *Ae*. *aegypti*)
**Diagnostic monitoring**	Deploy resistance data for threshold-based interventions.	- Molecular: *kdr* allele frequency qPCR (threshold: >60% → switch MoA) - Bioassay: WHO tube tests (mortality < 90% → avoid pyrethroids)
**Biological/genetic tools**	Exploit natural enemies or genetic mechanisms.	- Biocontrol: *Beauveria bassiana* GHA strain (EPF) against resistant *Bemisia tabaci* - Genetic: *Wolbachia* (wMel)-mediated population suppression in *Ae*. *aegypti*
**Policy & stewardship**	Enforce regulations to delay resistance.	- IRAC MoA classification: Mandatory rotation schedules per crop/pest - WHO GPIRM: National bans on agriculture-grade pyrethroids for malaria control

## Data Availability

Not applicable.

## Abbreviations

The following abbreviations are used in this manuscript:

AChE	Acetylcholinesterase
AI	Artificial Intelligence
*Bt*	*Bacillus thuringiensis*
CESs	Carboxylesterases (commonly abbreviated as CESs or CarEs)
CRISPR-Cas9	Clustered Regularly Interspaced Short Palindromic Repeats-CRISPR associated protein 9
CSPs	Chemosensory Proteins
CYPs	Cytochrome P450 Monooxygenases
DDT	Dichlorodiphenyltrichloroethane
DEM	Diethyl Maleate
dsRNA	Double-Stranded RNA
FAW	Fall Armyworm (*Spodoptera frugiperda*)
GABA	Gamma-Aminobutyric Acid
GPIRM	WHO Global Plan for Insecticide Resistance Management
GSTs	Glutathione-S-Transferases
HaNPV	*Helicoverpa armigera* Nucleopolyhedrovirus
IPM	Integrated Pest Management
IRM	Insecticide Resistance Management
kdr	Knockdown Resistance
LLINs	Long-Lasting Insecticidal Nets
lncRNAs	Long Non-Coding RNAs
MACE	Modified Acetylcholinesterase
miRNAs	MicroRNAs
MoA	Mode of Action
nAChRs	Nicotinic Acetylcholine Receptors
NPV	Nucleopolyhedrovirus
OBPs	Odorant-Binding Proteins
OP	Organophosphates
PBO	Piperonyl Butoxide
PIPs	Plant-Incorporated Protectants
qPCR	Quantitative Polymerase Chain Reaction
RDL	Resistance to Dieldrin
RNAi	RNA Interference
RyR	Ryanodine Receptor
SliNPV	*Spodoptera litura* Nucleopolyhedrovirus
VGSC	Voltage-Gated Sodium Channel
WHO	World Health Organization
